# How Web Professionals Perceive Web Accessibility in Practice: Active Roles, Process Phases and Key Disabilities

**DOI:** 10.1007/978-3-030-58796-3_35

**Published:** 2020-08-10

**Authors:** Beat Vollenwyder, Klaus Opwis, Florian Brühlmann

**Affiliations:** 8 Institute Integriert Studieren, JKU Linz, Linz, Austria; 9 Jack Baskin School of Engineering, UC Santa Cruz, Santa Cruz, CA USA; 10 Dipartimento di Meccanica, Politecnico di Milano, Milan, Italy; 11 Support Centre for Students with Special Needs, Masaryk University Brno, Brno, Czech Republic; Center for Cognitive Psychology and Methodology, Department of Psychology, University of Basel, Basel, Switzerland

**Keywords:** Web accessibility, Web professionals, Awareness, Survey

## Abstract

Providing usable web information and services to as many people as possible confronts web professionals with a challenging task. The present study delivers insights about how Web accessibility is perceived in practice. Using a survey, a total of 163 web professionals in various roles reported their evaluation of Web accessibility implementation in their projects with regard to three aspects: the professional roles primarily responsible for Web accessibility, key phases in the development process, and the types of disabilities primarily considered. Results show that non-technical professional roles are perceived to be less involved in the development process, that Web accessibility considerations are mainly restricted to the design and implementation phases of projects, and that efforts focus predominantly on the needs of people with visual impairments.

## Introduction

Web accessibility aims to provide usable web information and services to as many people as possible. It thereby contributes substantially to the web’s role in enabling and promoting equal participation in society [6]. However, web professionals face a challenging task when considering Web accessibility in their work. The first hurdle to overcome relates to its basic adoption in industrial practices. Here, it is essential that all organisational levels acquire a fundamental awareness and understanding of Web accessibility [12]. Insufficient knowledge gives rise to potentially harmful misconceptions about Web accessibility, such as beliefs that too few people benefit from it, that Web accessibility compromises aesthetics and technologically advanced solutions, or that the responsibility for addressing accessibility concerns lies solely in the hands of the developers [3, 6]. Consequently, Web accessibility issues are often deprioritised relative to other requirements [13]. In contrast, findings show that Web accessibility contributes to improved performance and perceived usability for all user groups [9].

The actual implementation of Web accessibility poses further challenges. To address the needs of users with disabilities, web professionals are required to gain a thorough understanding of how to design and implement accessible solutions for individual forms of sensory, motor and cognitive impairments. They need to consider not only how the various set-ups that are available for assistive technologies, such as screen readers, screen magnifiers and alternative input mechanisms [2, 11] can be employed, but also how they will be handled by people with different skill levels [14]. Further, web professionals are confronted with a large heterogeneity of constantly evolving web technologies. While this constitutes a major strength in terms of flexibility and adaptability, it can present an obstacle to the consideration and implementation of Web accessibility [5].

Several Web accessibility guidelines are available that provide information and advice on how to overcome these challenges. The Web Content Accessibility Guidelines (WCAG) are one of the most widely applied resources: They serve as the de facto standard for Web accessibility and are referenced in the policies of several countries [17]. However, despite the availability of the second edition of the WCAG since 2008 and their incorporation in legislation, adoption rates of Web accessibility standards still remain low. In February 2020, over 98% of the top million home pages on the web have detectable WCAG failures [18].

In a recent study, we aimed to identify the contributing factors underlying this neglect [15]. We identified three key determinants motivating web professionals to consider Web accessibility. First, users should actively communicate their specific needs. This can be facilitated by involving users with special needs in the development process. Second, web professionals should consider Web accessibility as an integral part of their professional role. They should become conscious of their responsibility to promote Web accessibility and ensure that expertise in implementing Web accessibility effectively is developed and supported. Third, Web accessibility should be perceived as beneficial for the overall quality of a product. This can be supported by promoting Web accessibility as a quality feature for all user groups.

As part of the aforementioned study [15], a subset of the participating web professionals were asked to comment on their evaluation of Web accessibility implementation practises in their own projects. These insights contribute to a deeper understanding of the professional roles that are primarily responsible for Web accessibility, the phases in the development process that are key to adoption and implementation, and the types of disabilities that are most often considered. The present paper reports these findings and discusses implications for Web accessibility research and practice.

## Method

### Participants and Design

An online survey was advertised via various computer science and human-computer interaction newsletters and mailing lists across Switzerland. The study description addressed, “all specialists who participate in the creation of websites and web applications (developers, designers, project managers, etc.)”. As an incentive, participants could take part in a lottery for two gift vouchers worth CHF 50.- (approximately EUR 46.-). A total of 166 participants completed the online survey, of which 163 participants (age *M* = 37.1, *SD* = 8.5, range 20–65; 53 women, 110 men) were included in the analysis. Three participants were removed from the sample, because their response time exceeded one hour. On average, the study took 16 min (*SD* = 8.5 min, range 2.5–56) to complete.

The majority of participants completed the questionnaire in German (*N* = 146), followed by French (9) and English (8). When describing their main role as web professional, most participants reported being employed in functional testing (32), management (29), user research and usability testing (14), product owner (13) visual design (13) and development (12). Compared to other employees at their organisation, the participants reported moderate business (*M* = 5.42, *SD* = 1.26; all items measured on a 7-point Likert-type rating scale, where 1 = *very low*, and 7 = *very high*) and technical expertise (*M* = 4.31, *SD* = 1.66) in web development. Similar ratings were reported regarding personal factors: Web accessibility interest (*M* = 5.48, *SD* = 1.27), knowledge (*M* = 4.85, *SD* = 1.51) and familiarity with WCAG (*M* = 4.59, SD = 1.59). Sixteen participants reported a disability, including visual (*N* = 9), hearing (2), motor (1) and cognitive (1), as well as unspecified (3) impairments.

Most participants reported working in large organisations with more than 250 employees (*N* = 81), followed by middle-sized organisations with 10 to 250 employees (52), and small organisations with under 10 employees (30). Organisations were active in the private sector (106), the public sector (57), science and education (35), trusts, societies or non-governmental organisations (26), and other domains (23). The main geographical regions of the organisations’ operations were Switzerland (134), Europe (14), global (13), and other individual or not further specified countries (2). Regarding legal obligations on Web accessibility, participants stated that a majority of their organisations were not obliged to consider Web accessibility (79), followed by organisations that were fully (38) and partially (10) obliged. A sizeable number of participants were not aware of the exact legal requirements of their organisation (36).

### Procedure

Participants were first asked to describe their professional role and to rate their business and technical expertise in web development. Further, participants were asked whether they were familiar with the term ‘Web accessibility’, which was a requirement for taking part in the present study. Participants who were neither aware nor informed about this issue were screened out and could not complete the questionnaire. Participants then rated their level of personal interest in Web accessibility, their knowledge about it, and their familiarity with WCAG. This was followed by questions regarding the size, domain, location and legal obligations of the participants’ organisation. Participants were also asked to report which professional roles (e.g., product owner, visual designer) were responsible for matters of Web accessibility in their organisation, the phases (e.g., analysis, design; see also [1]) involved in the development process and to report which types of disabilities (e.g., hearing impairments, motor impairments) were considered in their implementation efforts.

## Results

With regard to specifying the professional roles of the individuals in their organisation who hold primary responsibility for Web accessibility, participants listed professionals in interaction design (*N* = 80, 49.1%; multiple answers were possible), development (75, 46.0%), and user research and usability testing (68, 41.7%), followed by visual design (59, 36.2%) and dedicated accessibility experts (54, 33.1%). Professional roles in management (26, 16.0%) and functional testing (21, 12.9%) were mentioned the least. Full results are presented in Table 1.

**Table 1.** Professional roles primarily responsible for Web accessibility. Multiple answers were possible.

Key web professionals	N	%
Interaction design	80	49.1
Development	75	46.0
User research & usability testing	68	41.7
Visual design	59	36.2
Accessibility expert	54	33.1
Project management	46	28.2
Content specialist	42	25.8
Product owner	35	21.5
Business analysis	33	20.2
Management	26	16.0
Software architecture	26	16.0
Other	25	15.3
Functional testing	21	12.9

**Fig. 1.**
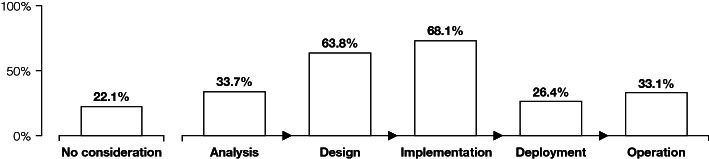
Bar diagram presenting the percentage values per phase, in which Web accessibility is considered during the development process. Multiple answers were possible.

With regard to identifying the phases in which Web accessibility is considered during the development process, participants mentioned the implementation (*N* = 111, 68.1%; multiple answers were possible) and design (104, 63.8%) phases most frequently, followed by analysis (55, 33.7%), operation (54, 33.1%), as well as the deployment phase (43, 26.4%). A substantial number of participants reported that Web accessibility is not considered at all in their organisation’s development process (36, 22.1%). A schematic representation of the results is presented in Fig. 1.

Participants reported that most of their organisation’s Web accessibility efforts focus on people with visual impairments (*M* = 5.23, *SD* = 1.89; all items measured on a 7-point Likert-type rating scale, where 1 = *very low importance*, and 7 = *very high importance*), blindness (*M* = 4.87, *SD* = 2.21), and motor impairments (*M* = 3.77, *SD* = 2.03), whereas deafness (*M* = 3.30, *SD* = 2.04), hearing impairments (*M* = 3.27, *SD* = 2.07), as well as learning and attention disorders (*M* = 3.09, *SD* = 1.91) receive less attention. All results are presented in Table 2.

**Table 2.** Participant responses rating the types of disabilities that are most often considered in Web accessibility efforts on a 7-point scale of ascending importance.

	M	SD
Visual impairments	5.23	1.89
Blindness	4.87	2.21
Motor impairments	3.77	2.03
Deafness	3.30	2.04
Hearing impairments	3.27	2.07
Learning and attention disorders	3.09	1.91

## Discussion

### Professional Roles Primarily Responsible for Web Accessibility

The role of the developer is identified as being one of the professional roles that is primarily responsible for considering Web accessibility. While this result was expected from previous insights [6], it is a more recent development to observe interaction design, user research and usability testing professionals emerge in this category. This may reflect discussions about the need for accessible solutions to go beyond the provision of basic access, and include aspects related to usability (e.g., effectiveness, efficiency and satisfaction) and user experience (e.g., affect, trust or aesthetics) [8]. Encouragingly, these results are in line with the strong agreement among accessibility experts that Web accessibility must be grounded in user-centred methodologies [19] and show that this understanding is in the process of being established in practice. Dedicated accessibility experts, however, were not reported among the top ranks. Even though most participants reported being part of a large organisation, accessibility experts may still be less common or less well known, and therefore receive less recognition than other roles. It may also indicate that accessibility experts are occupied with promoting a general awareness and understanding of Web accessibility, rather than being directly involved in development projects [12]. Finally, less technical professional roles, such as product owner, business analysis and management were rarely mentioned. This research corroborates previous studies in finding non-technical roles to be less aware of accessibility issues [4, 19]. The absence of concern for Web accessibility in non-technical roles may compromise the adoption strategies of their organisation, as these professionals are often in charge of prioritising or funding requirements.

### Key Phases in the Development Process

Web accessibility seems to be mostly considered in the design and implementation phases of the development process. While this may be appropriate for the development of a new product, few participants reported that Web accessibility was considered while adding new functionalities once the product was operational. Given that many products are often continuously improved after their initial launch, this poses a potential pitfall in ensuring the quality of web information and services as products continue to evolve [1]. For instance, if the login mechanism of a web service is updated and not tested for Web accessibility, it may block users from obtaining otherwise perfectly usable content. However, the present study did not include any questions concerning whether a product is developed iteratively, for instance, in applying an agile methodology. This may have influenced how participants rated the implementation and the subsequent development during operational phases. The issue of Web accessibility is also less prominent in the analysis phase, including during definition of the business case and risk analysis assessment. This may reflect the widespread belief that few people benefit from Web accessibility, and that it therefore provides only a limited competitive edge for a business [3]. It might also be closely tied to the aforementioned finding that non-technical and managerial professionals tend to be less involved in the provision of accessible web information and services. At worst, this lack of involvement and the resulting omission in the analysis phase may ultimately have a detrimental impact on people’s lives by excluding them from various spheres of private, social, economic or political life. Also, the neglect of such issues may have legal consequences for an organisation in the future [17].

### Types of Disabilities Primarily Considered

Most reported Web accessibility efforts are focused on visual impairments. Previous studies found that accessibility experts rated users with visual impairments as being an important target group, but not the only beneficiaries [19]. In contrast, the present findings reveal that there is a bias against users with non-visual impairments in practice. This bias may be due to the perception of the web as being a mainly visual medium and confirms widespread misconceptions that Web accessibility only benefits users with visual impairments [3]. In contrast, learning and attention disorders are only rarely considered. This is in line with ongoing discussions regarding the persisting need to reduce barriers for people with cognitive disabilities in current Web accessibility practice and policy [7]. Hence, it is important to highlight the beneficial effects of Web accessibility for all user groups, while potentially problematic aspects, such as contradictory findings regarding language complexity in the web, should be further explored to provide guidance for practice [10, 16].

## Conclusion

The present study provides insights into how web professionals evaluate the implementation of Web accessibility in practice. While there is evidence that user-centred practices in combination with Web accessibility are in the process of being established, non-technical web professionals tend to be less involved in supporting accessibility issues. Web accessibility is mainly considered in the design and implementation phases of the development process. The lack of attention to Web accessibility during the analysis phase and ongoing developments may result in missed opportunities and less accessible solutions after the product’s initial release. Future research should therefore explore how Web accessibility can be promoted among all professional roles and during the complete development process of a product. Further, results indicate that most Web accessibility efforts predominantly focus on people with visual impairments. It is therefore recommended to actively promote awareness and understanding of Web accessibility and to broaden the perspective at all organisational levels. We hope that these findings will guide further research activities and support web professionals in their quest to make the web more inclusive.

## References

[1] Abou-Zahra S, Harper S, Yesilada Y (2008). Web accessibility evaluation. Web Accessibility.

[2] Barreto A, Harper S, Yesilada Y (2008). Visual impairments. Web Accessibility.

[3] Ellcessor, E.: $$<$$ALT = “Textbooks”$$>$$: Web accessibility myths as negotiated industrial lore. Crit. Stud. Media Commun. **31**(5), 448–463 (2014).10.1080/15295036.2014.919660

[4] Freire AP, Russo CM, Fortes RPM (2008). The perception of accessibility in Web development by academy, industry and government: a survey of the Brazilian scenario. New Rev. Hypermedia Multimed..

[5] Harper S, Chen AQ (2011). Web accessibility guidelines. World Wide Web.

[6] Henry SL, Thatcher J (2006). Understanding web accessibility. Web Accessibility: Web Standards and Regulatory Compliance.

[7] Lewis C, Harper S, Yesilada Y (2008). Cognitive and learning impairments. Web Accessibility.

[8] Power C, Cairns P, Barlet M, Filimowicz M, Tzankova V (2018). Inclusion in the third wave: access to experience. New Directions in Third Wave Human-Computer Interaction: Volume 1 - Technologies.

[9] Schmutz S, Sonderegger A, Sauer J (2017). Implementing recommendations from web accessibility guidelines: a comparative study of nondisabled users and users with visual impairments. Hum. Factors: J. Hum. Factors Ergon. Soc..

[10] Schmutz S, Sonderegger A, Sauer J (2019). Easy-to-read language in disability-friendly web sites: effects on nondisabled users. Appl. Ergon..

[11] Trewin S, Harper S, Yesilada Y (2008). Physical impairment. Web Accessibility.

[12] Urban, M., Burks, M.R.: Implementing accessibility in the enterprise. In: Thatcher, J., et al. (eds.) Web Accessibility: Web Standards and Regulatory Compliance, pp. 69–83. Apress, Berkeley (2006). 10.1007/978-1-4302-0188-5_3

[13] Velleman EM, Nahuis I, van der Geest T (2015). Factors explaining adoption and implementation processes for web accessibility standards within eGovernment systems and organizations. Univ. Access Inf. Soc..

[14] Vigo M, Harper S (2014). A snapshot of the first encounters of visually disabled users with the web. Comput. Hum. Behav..

[15] Vollenwyder B, Iten GH, Brühlmann F, Opwis K, Mekler ED (2019). Salient beliefs influencing the intention to consider web accessibility. Comput. Hum. Behav..

[16] Vollenwyder B, Schneider A, Krueger E, Brühlmann F, Opwis K, Mekler ED, Miesenberger K, Kouroupetroglou G (2018). How to use plain and easy-to-read language for a positive user experience on websites. Computers Helping People with Special Needs.

[17] Waddell, C.D.: Worldwide accessibility laws and policies. In: Thatcher, J., et al. (eds.) Web Accessibility: Web Standards and Regulatory Compliance, pp. 547–579. Apress, Berkeley (2006). 10.1007/978-1-4302-0188-5_17

[18] WebAIM: The WebAIM Million, February 2019. https://webaim.org/projects/million. Accessed 12 Jun 2020

[19] Yesilada Y, Brajnik G, Vigo M, Harper S (2014). Exploring perceptions of web accessibility: a survey approach. Behav. Inf. Technol..

